# Removal of the C6 Vaccinia Virus Interferon-β Inhibitor in the Hepatitis C Vaccine Candidate MVA-HCV Elicited in Mice High Immunogenicity in Spite of Reduced Host Gene Expression

**DOI:** 10.3390/v10080414

**Published:** 2018-08-08

**Authors:** María Q. Marín, Patricia Pérez, Carmen E. Gómez, Carlos Óscar S. Sorzano, Mariano Esteban, Juan García-Arriaza

**Affiliations:** 1 Department of Molecular and Cellular Biology, Centro Nacional de Biotecnología (CNB), Consejo Superior de Investigaciones Científicas (CSIC), 28049 Madrid, Spain; mquiros@cnb.csic.es (M.Q.M.); pperez@cnb.csic.es (P.P.); cegomez@cnb.csic.es (C.E.G.); 2 Biocomputing Unit, Centro Nacional de Biotecnología (CNB), Consejo Superior de Investigaciones Científicas (CSIC), Madrid 28049, Spain; coss@cnb.csic.es

**Keywords:** HCV, poxvirus, MVA, vaccine, C6L, interferon, host gene expression, mice, cellular responses, humoral responses

## Abstract

Hepatitis C virus (HCV) represents a major global health problem for which a vaccine is not available. Modified vaccinia virus Ankara (MVA)-HCV is a unique HCV vaccine candidate based in the modified vaccinia virus Ankara (MVA) vector expressing the nearly full-length genome of HCV genotype 1a that elicits CD8^+^ T-cell responses in mice. With the aim to improve the immune response of MVA-HCV and because of the importance of interferon (IFN) in HCV infection, we deleted in MVA-HCV the vaccinia virus (VACV) *C6L* gene, encoding an inhibitor of IFN-β that prevents activation of the interferon regulatory factors 3 and 7 (IRF3 and IRF7). The resulting vaccine candidate (MVA-HCV ΔC6L) expresses all HCV antigens and deletion of *C6L* had no effect on viral growth in permissive chicken cells. In human monocyte-derived dendritic cells, infection with MVA-HCV ΔC6L triggered severe down-regulation of IFN-β, IFN-β-induced genes, and cytokines in a manner similar to MVA-HCV, as defined by real-time polymerase chain reaction (PCR) and microarray analysis. In infected mice, both vectors had a similar profile of recruited immune cells and induced comparable levels of adaptive and memory HCV-specific CD8^+^ T-cells, mainly against p7 + NS2 and NS3 HCV proteins, with a T cell effector memory (TEM) phenotype. Furthermore, antibodies against E2 were also induced. Overall, our findings showed that while these vectors had a profound inhibitory effect on gene expression of the host, they strongly elicited CD8^+^ T cell and humoral responses against HCV antigens and to the virus vector. These observations add support to the consideration of these vectors as potential vaccine candidates against HCV.

## 1. Introduction

Hepatitis C virus (HCV), a member of the genus *Hepacivirus*, family *Flaviviridae*, is an enveloped icosahedral virus, with positive-sensed and single-stranded RNA genome of 9600 nucleotide bases long [1]. HCV infection is a global health problem for which no vaccine is available. Upon HCV infection, 15–30% of people are able to clear the virus, while the remaining 70–85% will develop a chronic infection that can end with liver cirrhosis and hepatocellular carcinoma. It is estimated that 71 million people suffer from chronic hepatitis C and approximately 400,000 die every year as a result of HCV-related diseases, as reported by the World Health Organization (http://www.who.int/mediacentre/factsheets/fs164/en).

Recently approved direct-acting antiviral agents (DAAs) are able to cure HCV at high rates, but they still have important limitations such as the emergence of drug resistance variants, their high cost, safety issues especially in advanced chronic disease, viral genotype dependency, and the fact that most individuals who are chronically infected are unaware of their infection and thus unlikely to seek treatment [2,3]. Moreover, DAAs do not prevent future infections [4], hence the development of a prophylactic or therapeutic vaccine is a public health priority. Viral genome variability and immune evasion, lack of a suitable animal model, and unclear correlations of protection during HCV infection represent the main hurdles for the development of a successful vaccine [5]. However, spontaneous clearance of HCV infection and the ability to resolve subsequent infections in humans [6,7] and chimpanzees [8] yield important evidence of a protective immune response with a memory component, making a vaccine a feasible goal.

Current efforts in HCV vaccine development are aimed to induce strong T cell and humoral immune responses that are able to target the most conserved viral antigens without producing liver immunopathology. The importance of HCV-specific CD4^+^ and CD8^+^ T cell responses in the clearance of primary infection and reinfection has been reported [9,10,11]. Recent studies have also proven the key role of antibody response to E1 and E2 HCV glycoproteins in the resolution of HCV infection (reviews in the literature [11,12]). To date, several vaccine candidates against HCV have been tested with different rate of success [13]. Among them, poxvirus vectors and in particular, the modified vaccinia virus Ankara (MVA), possesses ideal properties as vaccines because of their ability to induce strong T and B immune responses, a safety profile, low cost, ease of manufacture and administration, and a low prevalence of anti-vector immunity in the global population [14,15,16,17].

However, more efficient and optimized vaccine candidates able to enhance both cellular and humoral immune responses against HCV antigens are desirable. One of the strategies is to enhance the induction of interferon (IFN), which is essential to trigger a potent defense against intracellular pathogens, such as HCV, and create an antiviral state in surrounding cells that will help to avoid the transition from the acute to the chronic phase of infection [18,19]. Additionally, one of the main approaches that has been used to improve the immunogenicity of MVA recombinants is the deletion of immunosuppressive genes that are still present in the MVA genome [20]. One of these vaccinia virus (VACV) immunomodulatory genes is *C6L*, encoding the C6 protein, a non-essential virulent factor expressed early in infection [21,22]. C6 interacts with the scaffold proteins TANK, NAP1, and SINTBAD and inhibits the kinases TBK1 and IKKε, which are the activators of the transcription factors IRF3 and IRF7, resulting in a blockade of IFN-β expression [21]. Moreover, C6 inhibits type I IFN signaling in the nucleus and binds to the transactivation domain of STAT2 [23]. Deletion of VACV *C6L* gene in the HIV/AIDS vaccine candidate MVA-B enhanced HIV-1-specific cellular and humoral immune responses in mice in comparison with the parental MVA-B vector without deletions, and induced the expression of type I IFN and IFN-α/β inducible genes in human macrophages and monocyte-derived dendritic cells (moDCs) [22,24]. Moreover, vaccination with the VACV strain Western Reserve (WR), lacking the *C6L* gene, provided better protection against a challenge with a lethal dose of WR, and induced an enhanced immunogenicity [25].

We have previously described a vaccine candidate against HCV based on MVA strain constitutively expressing the nearly full-length HCV genome from genotype 1a (termed MVA-HCV). In vaccinated mice, MVA-HCV induced polyfunctional HCV-specific CD8^+^ T cell immune responses, mainly directed against p7 + NS2 and NS3. Moreover, MVA-HCV induced memory T cell responses with an effector memory phenotype [26]. With the purpose to enhance the immune responses of MVA-HCV, we reasoned that similar to what we have previously observed of immune improvements with an HIV/AIDS vaccine (MVA-B) lacking the *C6L* gene, the same deletion might help to increase the immune responses induced by the MVA-HCV vaccine candidate. To this aim, we deleted the VACV *C6L* gene in MVA-HCV, coding for an inhibitor of IFN-β, and performed a head-to-head comparison between MVA-HCV and MVA-HCV ΔC6L, analyzing the expression of HCV proteins and evaluating, by real-time polymerase chain reaction (PCR) and microarrays, the profile of host gene expression induced after infection of human moDCs or macrophages. Furthermore, we have analyzed the innate immune responses in mice inoculated with MVA-HCV and MVA-HCV ΔC6L, together with the adaptive and memory HCV-specific T cell and humoral immune responses in vivo. Our findings revealed that both MVA-HCV vectors are capable of activating HCV and vector-specific CD8^+^ T cell and humoral immune responses in spite of the suppressive transcriptional effects mediated by HCV proteins.

## 2. Materials and Methods

### 2.1. Ethics Statement

The performed mouse experiments were approved by the Ethical Committee of Animal Experimentation (CEEA) of Centro Nacional de Biotecnología (CNB, Madrid, Spain) according to international guidelines and the Spanish law under the Royal Decree (RD 53/2013) (permit number PROEX 331/14; 30 January 2015). Animals were maintained and handled at the CNB in a pathogen-free animal facility, following the Federation of European Laboratory Animal Science Associations recommendations. Human buffy coats from healthy blood donors were provided by the Centro de Transfusion de la Comunidad de Madrid (Madrid, Spain) and their use was approved by their Ethical Committee.

### 2.2. Cells and Viruses

The established DF-1 cells (an immortalized chicken embryo fibroblast (CEF) cell line), and primary cultures of CEF cells (obtained from 11-day-old eggs; Intervet, Salamanca, Spain) were grown in Dulbecco’s modified Eagles medium (DMEM) supplemented with 10% fetal calf serum (FCS) (Gibco-Life Technologies, Carlsbad, CA, USA), as previously described [26]. Human monocytic THP-1 cells were grown in complete Roswell Park Memorial Institute (RPMI) 1640 medium supplemented with 10% FCS, and were differentiated into macrophages 24 h before usage by treatment with 0.5 mM phorbol 12-myristate 13-acetate (PMA; Sigma-Aldrich, St. Louis, MO, USA), as previously described [22,24]. Freshly isolated peripheral blood mononuclear cells (PBMCs) from human buffy coats were obtained by Ficoll gradient separation on Ficoll–Paque (GE Healthcare, Chicago, IL, USA). Thereafter, monocytes were isolated and differentiated into moDCs, as previously described [22,24]. Cells were cultured at 37 °C in a humidified incubator containing 5% CO_2_.

The vaccine poxviruses used in this study were the wild-type attenuated MVA (MVA-WT), and the recombinant MVA-HCV that expresses the nearly full-length HCV genome (proteins Core, E1, E2, p7, NS2, NS3, NS4A, NS4B, NS5A, and a part of NS5B; genotype 1a), which are inserted into the MVA thymidine kinase (TK) locus under the control of the viral synthetic early/late (sE/L) promoter [26]. The recombinant virus MVA-HCV was used as the parental virus for the generation of the modified recombinant MVA-HCV ΔC6L (see later). Virus infections in cells were performed as previously described [27,28]. Viruses were grown in primary CEF cells, purified by two-step sucrose cushions, and titrated in DF-1 cells by plaque immunostaining assay, as previously described [29]. All viruses were free of contamination with either mycoplasma (checked by specific PCR test), bacteria (checked by growth in LB plates without ampicillin), or fungi (checked by growth in Columbia blood agar plates; Oxoid, Waltham, MA, USA).

### 2.3. Plasmid Transfer Vector pGem-RG-C6L wm

The plasmid transfer vector pGem-RG-C6L wm was previously generated [22] and directs the deletion of VACV *C6L* gene from the MVA-HCV genome. The gene *MVA 019L* in MVA is equivalent to *C6L* in VACV Copenhagen strain, and for simplicity, we used the open reading frame nomenclature of the VACV Copenhagen strain to refer the MVA genes throughout this manuscript.

### 2.4. Construction of MVA-HCV ΔC6L

MVA-HCV ΔC6L recombinant virus, containing a deletion of the VACV gene *C6L*, was generated by homologous recombination in DF-1 cells after infection with MVA-HCV follow up by transfection with the plasmid pGem-RG-C6L wm. The infection/transfection was then screened for transient Red2/GFP co-expression using dsRed2 and rsGFP genes as selectable markers, as previously described [22,30]. The isolated MVA-HCV ΔC6L recombinant virus was obtained after six consecutive rounds of plaque purification and confirmation of the loss of selectable markers.

### 2.5. PCR Analysis

The precise generation and purity of MVA-HCV ΔC6L was checked by PCR with primers TK-L and TK-R, annealing in the VACV TK locus for the amplification of the HCV genes, and primers RFC6L-AatII-F and LFC6L-BamHI-R, annealing in the VACV *C6L* flanking regions for the detection of the *C6L* deletion, as previously described [22,26]. All the amplification reactions were performed with Phusion^®^ High-Fidelity DNA Polymerase (New England Biolabs, Ipswich, MA, USA) according to the manufacturer’s recommendations. The 7868 Kb HCV insertion and the *C6L* deletion in the genome of the recombinant viruses were also confirmed by DNA sequence analysis (Macrogen, Seoul, South Korea).

### 2.6. Expression of HCV Proteins

The correct expression of HCV proteins by MVA-HCV ΔC6L was checked by Western blotting from cells extracts obtained from monolayers of DF-1 cells mock infected or infected with five plaque-forming units (PFU)/cell with MVA-WT, MVA-HCV, or MVA-HCV ΔC6L. At 24 h post-infection (hpi), cells were recovered by scraping and centrifuged at 3000 rpm for 5 min; cellular pellets were lysed in Laemmli 1× + β-mercaptoethanol solution; and the proteins were fractionated by 10% SDS-PAGE and analyzed by Western blotting with mouse polyclonal antibody against HCV Core (C7-50; diluted 1:5000), mouse monoclonal antibodies against E1 (Acris Antibodies, Herford, Germany; diluted 1:1000), E2 (GenWay Biotech, Pretoria, Southafrica; diluted 1:1000), and NS5A (GenWay Biotech, diluted 1:1000) or goat polyclonal antibody against NS3 (Abcam, Cambridge, UK; diluted 1:500), to assess the expression of the different HCV proteins. Rabbit anti-β-actin (Cell Signaling, Danvers, MA, USA; diluted 1:1000) and rabbit anti-VACV E3 (Centro Nacional de Biotecnología, Madrid, Spain; diluted 1:1000) antibodies were used to define loading controls. An anti-rabbit-horseradish peroxidase (HRP)-conjugated antibody (Sigma; diluted 1:5000), anti-mouse-HRP-conjugated antibody (Sigma; diluted 1:2000), or anti-goat-HRP-conjugated antibody (Sigma; diluted 1:5000) were used as secondary antibodies. The immunocomplexes formed were visualized using an HRP-luminol enhanced chemiluminescence system (ECL Plus; GE Healthcare).

### 2.7. Analysis of Virus Growth

To study the virus growth profile of MVA-HCV ΔC6L, in comparison with parental MVA-HCV, monolayer cultures of DF-1 cells were infected at 0.01 PFU/cell with MVA-HCV or MVA-HCV ΔC6L, as previously described [22,24]. At 0, 24, 48, and 72 hpi, cells were collected and virus titers in cell lysates were analyzed by a plaque immunostaining assay, as previously described [29].

### 2.8. RNA Analysis by Quantitative Real-Time PCR

Human THP-1 cells or moDCs were mock infected or infected at 5 PFU/cell (for THP-1 cells) or at 0.3 and 1 PFU/cell (for moDCs) with MVA-WT, MVA-HCV, or MVA-HCV ΔC6L, and total RNA was isolated at 6 hpi using the RNeasy kit (Qiagen, Hilden, Germany). Reverse transcription of 1000 ng of RNA was done using the QuantiTect Reverse Transcription kit (Qiagen), and quantitative real-time PCR was performed with a 7500 Real-Time PCR System (Applied Biosystems, Foster City, CA, USA) using the Power SYBR Green PCR Master Mix (Applied Biosystems), as previously described [27]. IFN-β, interferon-induced protein with tetratricopeptide repeats 1 and 2 (IFIT1, IFIT2) and tumor necrosis factor alpha (TNF-α) gene expression was calculated relative to the expression of hypoxanthine-guanine phosphoribosyltransferase (HPRT) in arbitrary units (A.U.), and was measured using specific oligonucleotides (their sequences are available upon request). Samples were analyzed in duplicate, and two independent experiments were performed.

### 2.9. Microarray Analysis

Human moDCs from three different healthy donors were mock infected or infected at 1 PFU/cell with MVA-WT, MVA-HCV, or MVA-HCV ∆C6L. At 6 hpi, total RNA was isolated with TRIzol (Thermo Fisher) following manufacturer’s recommendations. The three biological replicates were independently hybridized for each transcriptomic comparison. The one-color microarray-based gene expression analysis protocol (Agilent Technologies, Palo Alto, CA, USA) was used to amplify and label RNA. Briefly, 200 ng of total RNA was reverse transcribed using T7 promotor primer and the cDNA was converted to RNA using the T7 RNA polymerase enzyme, which simultaneously amplifies target material and incorporates cyanine 3-labeled CTP. Cy3 probes to the amount of 1650 ng were mixed and hybridized to a human oligo microarray 4 × 44 K (G2519F_026652, Agilent Technologies, Santa Clara, CA, USA) for 17 h at 65 °C in gex hybridization buffer HI-RPM in a hybridization oven. Arrays were washed according to the manufacturer’s instructions, dried by centrifugation, and scanned at 3 mm resolution on Agilent DNA microarrays scanner (G2565BA, Agilent Technologies), and the images were analyzed with feature extraction software (Agilent Technologies). Background correction and normalization of expression data were performed using LIMMA [31,32]. LIMMA is part of bioconductor, an R language project [33]. For the consideration of local background correction and normalization, the methods “normexp” and loess, respectively, in LIMMA were used [31]. To have a similar distribution across arrays and to achieve consistency among arrays, log-ratio values were scaled using the median-absolute-value as scale estimator [31].

Differentially expressed human genes were evaluated by the non-parametric algorithm “Rank Products” available as “RankProd” package at Bioconductor [34,35]. This method detects genes that are consistently highly ranked in a number of replicated experiments independently of their numerical intensities. The results are provided in the form of *p*-values defined as the probability that a given human gene is ranked in the observed position by chance. The expected false discovery rate was controlled to be less than 5%. Hybridizations and statistical analysis were performed by the Genomics Facility at Centro Nacional de Biotecnología, Madrid, Spain.

For microarray validation, the three donor RNA samples were reverse transcribed and used for real-time PCR to determine the expression levels of IFN-β, IFIT1, and IFIT2 in a similar way as described above.

### 2.10. Recruitment of Immune Cells in the Peritoneal Cavity of C57BL/6 Mice

C57BL/6JOlaHsd mice (six to eight weeks old) were purchased from Envigo (Huntingdon, UK). Groups of mice (*n* = 5) were injected intraperitoneally (i.p.) with 10^7^ PFUs per mouse of MVA-WT, MVA-HCV, MVA-HCV ∆C6L, or PBS. At 6, 24, and 48 hpi, peritoneal exudate cells were collected in 6 mL of PBS-2% FCS and the presence of different immune cells was defined by flow cytometry after staining with specific antibodies, as previously described [36]. We used the following antibodies: CD11c-FITC (clone N418; Bioscience; 1:50), Ly6G-PE (clone 1A8; BD; 1:100), CD11b-PE-Cy7 (clone M1/70; BD; 1:100), F4/80-APC (clone BM8; eBioscience; 1:50), CD19-APC-Cy7 (clone 1D3; BD; 1:100), MHC II (1-A/1-E biotin) (clone 2G9; BD; 1:100) + Avidin-PB (Invitrogen; 1:100), CD45-BV570 (clone 30-F11; Biolegend; 1:100), CD4-PE (clone GK1.5; BD; 1:100), CD8-PE-Cy7 (clone 53-6.7; BD; 1:200), NKp46-APC (clone 29A1.4; Biolegend; 1:100), and CD3-APC-Cy7 (clone 145-2C11; BD; 1:50).

Cells were acquired using a Gallios flow cytometer (Beckman Coulter, Pasadena, CA, USA) and data were analyzed using the FlowJo software (version 8.5.3; Tree Star, Ashland, OR, USA). The different immune cells analyzed (gated on CD45^+^ cells) were the following: dendritic cells (DCs) (CD11c^+^/MHC-II^+^), neutrophils (CD11b^+^/Ly6G^+^), neutrophils α (CD11b^med^/Ly6G^med^), neutrophils β (CD11b^high^/Ly6G^high^), macrophages (F4/80^high^/CD11b^high^), B cells (CD19^+^), B cells high (CD19^+^/CD11b^+^), CD8 T cells (CD3^+^/CD8^+^), CD4 T cells (CD3^+^/CD4^+^), natural killer (NKs) cells (NKp46^+^/CD3^−^), and NKT cells (NKp46^+^/CD3^+^).

### 2.11. Peptides

Purified HCV peptide pools of the HCV virus H77 strain (genotype 1a) were obtained through Biodefense and Emerging Infectious Research Resources Repository (BEI Resources; National Institute of Allergy and Infectious Disease, National Institutes of Health, Bethesda, MD, USA). Peptides cover the entire HCV H77 genome as consecutive 13- to 19-mers overlapping by 11 or 12 amino acids. Peptides were resuspended to a final concentration of 1 mg/mL and grouped into seven pools: core pool (28 peptides), E1 pool (28 peptides), E2 pool (55 peptides), p7 + NS2 pool (40 peptides), NS3 pool: comprising NS3-1 (49 peptides) plus NS3-2 (49 peptides), NS4 pool (47 peptides), and NS5 pool: comprising NS5-1 (55 peptides) plus NS5-2 (53 peptides), and NS5-3 (53 peptides). Peptides were used for ex vivo stimulation of splenocytes from immunized mice. The VACV peptide within the viral E3 protein (VGPSNSPTF) was used to analyze the induction of VACV-specific CD8^+^ T cell responses.

### 2.12. C57BL/6 Mice Immunization Schedule

Female C57BL/6JOlaHsd mice (six to eight weeks old) were acquired from Envigo. An MVA prime/MVA boost immunization protocol was performed to study the immunogenicity of MVA-HCV and MVA-HCV ∆C6L vaccine candidates. Mice of each group (*n* = 8) were immunized with 10^7^ PFU/mouse of MVA-HCV, MVA-HCV ∆C6L, or MVA-WT by i.p. route in 200 µL of PBS and two weeks later, they received a second dose of 10^7^ PFU/mouse of the same virus as in the prime. At 10 and 53 days after the last immunization, four mice in each group were sacrificed and spleens were processed to measure by intracellular cytokine staining (ICS) assay the adaptive and memory HCV-specific T cell immune responses, respectively. Furthermore, serum samples were obtained at the same time points to analyze the HCV-specific humoral immune responses. Moreover, the VACV-specific T cell and antibody responses to the three virus vectors were also analyzed. Four independent experiments were performed.

### 2.13. ICS Assay

The magnitude, breath, polyfunctionality, and phenotype of the HCV-specific T cell responses were analyzed by ICS, as previously described [22,24,26,30,37]. After spleen disruption, 4 × 10^6^ splenocytes were treated with NH_4_Cl 0.1 M in ice for 5 min to deplete the red blood cells. Then, splenocytes were seeded on 96-well plates and stimulated for 6 h in complete RPMI 1640 medium supplemented with 10% FCS containing 1 μL/mL of GolgiPlug (BD Biosciences, Franklin Lakes, N.J., USA), monensin 1× (eBioscience, Waltham, MA, USA), anti-mouse CD107a-Alexa Fluor^®^ 488 (clone 1D4B; eBioscience; 1:300), and 1 μg/mL of the different HCV peptide pools (Core, E1, E2, p7 + NS2, NS3, NS4, and NS5) or the VACV E3 peptide. Then, cells were washed with PBS 1×-2% FCS-2 mM EDTA (FACS Buffer), stained using 0.5 μL/mL of LIVE/DEAD™ Fixable Violet Dead Cell Stain Kit (Invitrogen, Carlsbad, CA, USA) for 30 min at 4 °C, and washed once with FACS Buffer. Cells were then stained for 20 min at 4 °C in FACS buffer containing the following fluorochrome-conjugated antibodies against different cell surface markers: PE-CF594 hamster anti-mouse CD3e (clone 145-2C11; BD; 1:100), APC-Cy™7 rat anti-mouse CD4 (clone GK1.5; BD; 1:100), V500 rat anti-mouse CD8a (clone 53-6.7; BD; 1:100), Alexa Fluor^®^ 700 rat anti-mouse CD62L (clone MEL-14; BD; 1:100), and PerCP-Cy5.5 rat anti-mouse CD127 (clone A7R34; eBioscience; 1:100). After one wash, the cells were fixed and permeabilized for 20 min at 4 °C using the Cytofix/Cytoperm™ kit (BD), followed by two washes in perm/wash buffer and addition of purified rat anti-mouse CD16/CD32 (Mouse Fc block™; clone 2.4G2; BD; 1:100) for 15 min at 4 °C. Next, cells were stained intracellularly for 20 min at 4 °C in perm/wash buffer containing the following fluorochrome-conjugated antibodies: PE-Cy™7 rat anti-mouse IFN-γ (clone XMG1.2; BD; 1:100), PE rat anti-mouse TNFα (clone MP6-XT22; eBioscience; 1:100), and APC rat anti-mouse IL-2 (clone JES6-5H4; BD; 1:40). Following staining, cells were washed twice in perm/wash buffer and finally resuspended in FACS buffer. Furthermore, cells that were stained individually with each fluorochrome-conjugated antibody were used as controls for compensation. Cells were acquired using a Gallios flow cytometer (Beckman Coulter) and flow cytometry data was analyzed using FlowJo software (version 8.5.3; Tree Star, Ashland, OR, USA). The background responses in negative-control samples (stimulated only with RPMI medium) were subtracted from those detected in stimulated samples for every specific functional combination.

### 2.14. Enzyme-Linked Immunosorbent Assay (ELISA)

Total IgG levels of binding antibodies to HCV E2 protein and to VACV proteins in serum samples from immunized mice were assessed by ELISA. Ninety-six-well plates (Nunc MaxiSorp^®^) were coated and left overnight at 4 °C with 2 μg/mL of purified recombinant HCV E2 protein (genotype 1a, isolate H77; Sino Biological, Wayne, PA, USA) or with 10 μg/mL of a soluble extract of BSC-40 cells infected (5 PFU/cell) for 24 h with VACV WR virus strain. Then, plates were washed three times with PBS 1× supplemented with 0.05% Tween-20 and blocked with 200 μL of PBS-5% milk-0.05% Tween-20 for 2 h at room temperature (RT). Next, serum samples were diluted 1:100 in PBS-1% milk-0.1% Tween-20, and 100 μL was added to the plates following an incubation for 1.5 h at RT. Then, plates were washed three times with PBS-0.05% Tween-20, and 100 μL of secondary HRP-conjugated goat anti-mouse IgG antibody (SouthernBiotech, Birmingham, AL, USA, diluted 1:1000 in PBS-1% milk-0.1% Tween-20) was added and incubated for 1 h at RT. After incubation, plates were washed three times with PBS-0.05% Tween-20, and 100 μL of 3,3′,5,5′-tetramethylbenzidine (TMB) (Life Technologies, Carlsbad, CA, USA) was added. After 15 min, 50 μL of H_2_SO_4_ 1 M was added to stop the colour development. Absorbance was read at 450 nm.

### 2.15. Statistical Procedures

Significant differences (* = *p* ≤ 0.05; ** = *p* ≤ 0.01; *** = *p* ≤ 0.001) in RT-PCR data were calculated using the Holm–Sidak method, with alpha = 5%. Differentially expressed genes in microarray analysis were selected after application of the following statistical filters (False discovery rate Prod *p* value < 0.05 or *p* value LIMMA < 0.05, and fold change ≥2 or ≤−2). The comparison between MVA-HCV and MVA-HCV ΔC6L in the experiment of cell recruitment was analyzed by a two-way analysis of variance (ANOVA), evaluating the significant differences considering viruses (regardless of time), time (regardless of viruses), and the interaction between viruses and time. The statistical analysis of the obtained ICS data was performed as previously described [30,38].

## 3. Results

### 3.1. Generation of MVA-HCV ΔC6L Deletion Mutant

**Figure 1:**
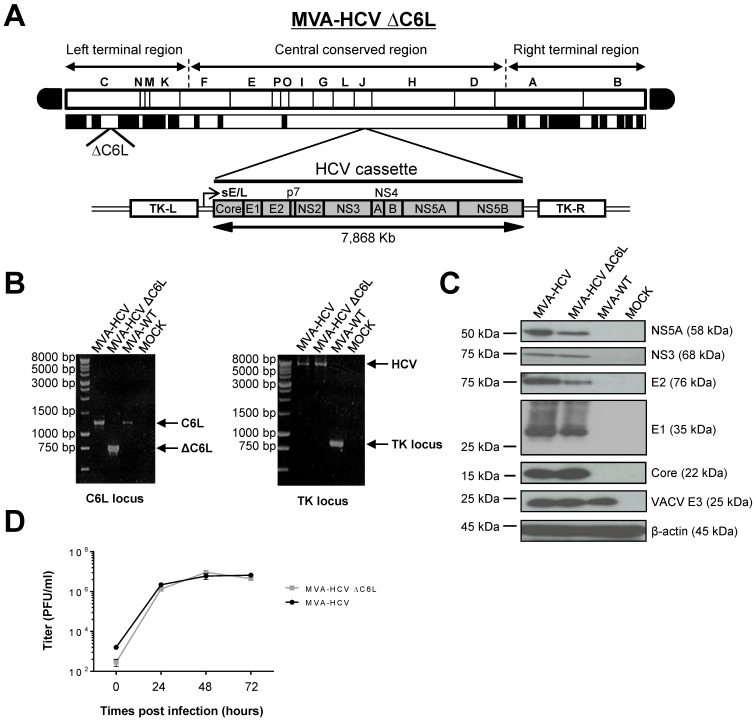
Generation and in vitro characterization of modified vaccinia virus Ankara (MVA)-hepatitis C virus (HCV) ΔC6L. (**A**) Scheme of the MVA-HCV ΔC6L deletion mutant genome map. The different regions of the MVA vector are shown in capital letters. Below the map, the deleted or fragmented vaccinia virus (VACV) genes are depicted as black boxes, with the deletion of *C6L* being indicated. The organization of the HCV genome (genotype 1a, strain H77), driven by the sE/L VACV promoter and inserted within the VACV thymidine kinase (TK) viral locus (J2R), is indicated. TK-L, TK left; TK-R, TK right. (**B**) Polymerase chain reaction (PCR) analysis of the VACV *C6L* (left panel) and TK (right panel) loci. DNA products are indicated by an arrow on the right. (**C**) Expression of HCV proteins in DF-1 cells infected with MVA-HCV or MVA-HCV ΔC6L at 24 h post-infection (hpi). (**D**) Viral growth kinetics in DF-1 cells infected with MVA-HCV or MVA-HCV ΔC6L. At different times post-infection, cells were harvested and virus titers in cell lysates were determined by plaque immunostaining assay with anti-VACV antibodies. The mean and standard deviation (SD) from two independent experiments are shown. WT—wild-type; PFU—plaque-forming units.

We have previously described the fact that deletion of the immunomodulatory VACV *C6L* gene, encoding an inhibitor of IFN-β [21,23], in the vector backbone of an MVA-based HIV/AIDS vaccine candidate induced the production of IFN-β and type I IFN inducible genes in infected innate immune cells and elicited an enhancement in the HIV-specific immunogenicity in immunized mice [22,24]. Thus, to examine whether VACV *C6L* gene could also influence the immunogenicity profile of HCV antigens delivered from a poxvirus vector, we deleted *C6L* from the vector backbone of the HCV vaccine candidate MVA-HCV (expressing the nearly full-length genome from HCV genotype 1a) [26], generating the recombinant MVA-HCV ΔC6L deletion mutant (Figure 1A), as described in Materials and Methods.

The correct generation (scheme in Figure 1A) and purity of MVA-HCV ΔC6L was confirmed by PCR, as it is shown in Figure 1B. Viral DNA was purified from DF-1 cells infected with MVA-HCV ΔC6L (or parental MVA-HCV and MVA-WT, used as controls) and amplified by PCR using a set of primers annealing in the *C6L* flanking regions, to confirm the presence of the deletion in the VACV *C6L* gene. The results showed that MVA-HCV ΔC6L has correctly deleted the VACV *C6L* gene (Figure 1B, left). Moreover, we confirmed the correct insertion of the HCV genes by PCR analysis using a set of primers annealing in the VACV TK flanking sequences, with MVA-HCV and MVA-HCV ΔC6L with a full-length size of about 8 Kb (Figure 1B, right), which was also confirmed by DNA sequencing of the inserted HCV genome. Thus, the MVA-HCV ΔC6L vector was successfully generated, with deletion of the VACV *C6L* gene and insertion of HCV genes in the MVA TK locus, and without the presence of wild-type virus.

### 3.2. Expression of HCV Proteins by MVA-HCV ΔC6L

To confirm that MVA-HCV ΔC6L constitutively expresses and successfully processes the HCV polyprotein, cultures of DF-1 cells were infected at 5 PFU/cell for 24 h with MVA-HCV ΔC6L, MVA-HCV, or MVA-WT, and protein expression of HCV Core, E1, E2, NS3, and NS5A proteins was resolved by Western blot. As shown in Figure 1C, the HCV open reading frame was efficiently transcribed and translated into viral proteins during MVA-HCV ΔC6L infection, as in the case of parental MVA-HCV; as such, a viral HCV polyprotein is properly processed into mature structural (Core, E1 and E2) and nonstructural (p7-NS2-NS3-NS4-NS5) HCV proteins.

### 3.3. VACV C6L Gene Is Non-Essential for MVA-HCV Growth

We have previously shown that MVA-HCV and MVA-WT grow similarly in cell culture [26]. Here, to further determine whether deletion of VACV *C6L* gene affects virus replication in cell culture, we next compared the virus growth of MVA-HCV ΔC6L and MVA-HCV in DF-1 cells. The results showed that the kinetics of viral growth are similar between parental MVA-HCV and MVA-HCV ΔC6L deletion mutant (Figure 1D), indicating that the *C6L* gene deletion does not impair virus replication under permissive conditions, and *C6L* is not required for MVA-HCV replication. Furthermore, the mere isolation of MVA-HCV ΔC6L deletion mutant demonstrates that the VACV C6 protein is not essential for MVA-HCV replication.

### 3.4. RT-PCR Showed that MVA-HCV and MVA-HCV ΔC6L Downregulate Expression of Genes Involved in Innate Immunity

**Figure 2:**
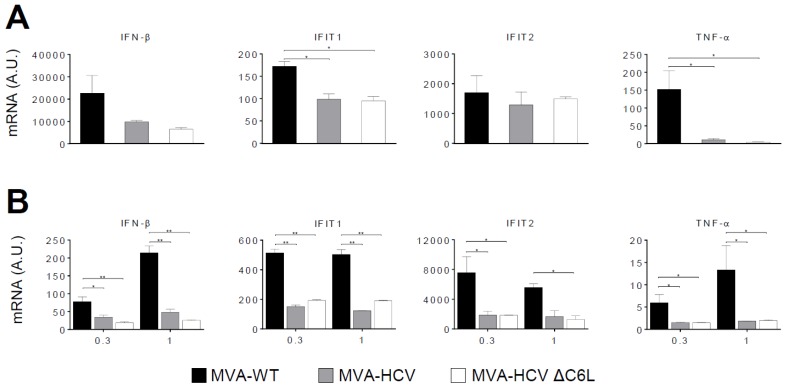
Innate immune responses induced by MVA-HCV and MVA-HCV ΔC6L in human macrophages and monocyte-derived dendritic cells (moDCs). Human THP-1 (human monocytic cell line derived from an acute monocytic leukemia patient) differentiated to macrophages (**A**) and moDCs (**B**) were mock infected or infected with MVA-WT, MVA-HCV, or MVA-HCV ΔC6L (5 PFU/cell in panel A and 0.3 or 1 PFU/cell in panel B). At 6 hpi, RNA was extracted and interferon (IFN)-β, IFN-β-induced gene (IFIT)1, IFIT2, tumor necrosis factor alpha (TNF-α), and hypoxanthine-guanine phosphoribosyltransferase (HPRT) mRNA levels were analyzed by RT-PCR. Results are expressed as the ratio of the gene to HPRT mRNA levels. A.U., arbitrary units. *p* values indicate significant response differences when comparing MVA-HCV and MVA-HCV ΔC6L with MVA-WT (* *p* < 0.05, ** *p* < 0.005). Data are means ± SD of duplicate samples from one experiment and are representative of two independent experiments.

We have previously reported that infection of cells with MVA-HCV reduced the expression of several genes involved in innate immunity compared with MVA-WT [26]. Thus, to examine whether C6 VACV type I IFN inhibitor protein is able to impair the response of innate immune cells to MVA-HCV, promoting an enhancement in the production of type I IFN, we analyzed the expression of type I IFN (IFN-β), IFN-β-induced genes (IFIT1 and IFIT2), and the proinflammatory cytokine TNF-α, by real-time PCR in human THP-1 cells (Figure 2A) or moDCs (Figure 2B) mock infected or infected for 6 h with MVA-WT, MVA-HCV, and MVA-HCV ΔC6L. The results showed that, compared with MVA-WT, the recombinant viruses MVA-HCV and MVA-HCV ΔC6L similarly down-regulated the expression of IFN-β, IFIT1, IFIT2, and TNF-α in both human cell types without significant differences between both vectors, while mRNA levels were upregulated by MVA-WT.

### 3.5. Microarray Analysis Revealed a Severe Reduction of Host Gene Expression by MVA-HCV and MVA-HCV ΔC6L

**Figure 3:**
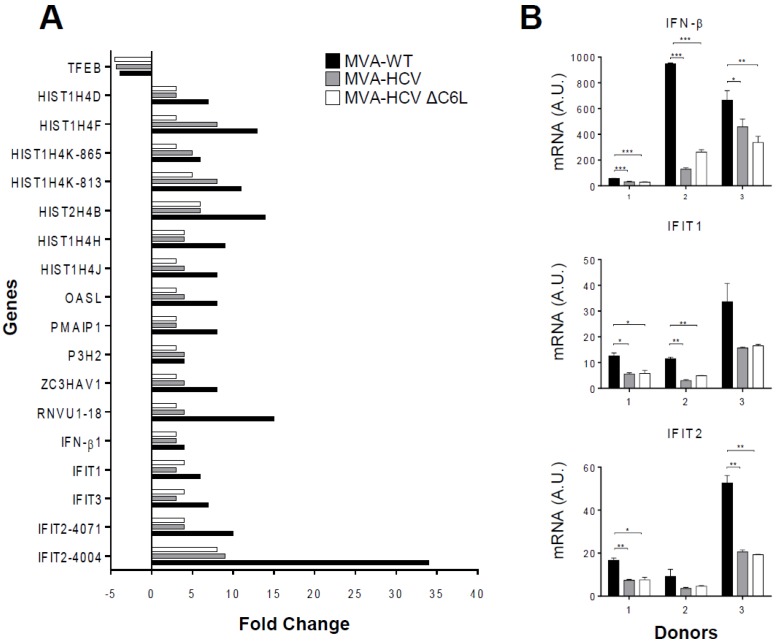
Microarray analysis of HCV antigen-regulated host genes in human moDCs infected with MVA-HCV and MVA-HCV ∆C6L. (**A**) Clustering classification of host genes differentially regulated in MVA-WT-, MVA-HCV-, and MVA-HCV ΔC6L-infected human moDCs versus mock infected cells (FDR Prod *p* value < 0.05, fold change ≥2 or ≤−2). MoDCs were infected for 6 h and the expression levels of different host genes were analyzed using a human oligo microarray 4 × 44 K, as described in Material and Methods. Differentially expressed genes were evaluated by the non-parametric algorithm “Rank Products” available as “RankProd” package at Bioconductor. Results are the mean obtained from three different healthy blood donors. (**B**) Validation of microarray data by real-time RT-PCR to determine the mRNA levels of IFN-β, IFIT1, and IFIT2 genes. RNA isolated from the three healthy blood donors used for microarray analysis was used. A.U., arbitrary units. Data are means ± SD of duplicate samples. *p* values indicate significant response differences comparing MVA-HCV and MVA-HCV ΔC6L with MVA-WT (* *p* < 0.05, ** *p* < 0.005, *** *p* < 0.001).

To further define whether the two MVA-HCV vectors have a similar or different impact on host gene expression, we performed a microarray analysis to study the differentially expressed host genes in moDCs obtained from three human donor samples and mock infected or infected with MVA-HCV, MVA-HCV ∆C6L, and MVA-WT at 1 PFU/cell after 6 h of infection. Under stringent conditions for the microarray analysis and compared with mock infected cells, the results showed that 17 host genes were significantly upregulated in moDCs infected with MVA-WT (Figure 3A, black bars). In the case of cells infected with MVA-HCV or MVA-HCV ∆C6L, host gene expression was still upregulated, but reduced several-fold in most of the genes when compared with MVA-WT (Figure 3A, grey and white bars); and only one gene (TFEB) was downregulated by the three vectors (Figure 3A). Genes significantly downregulated in MVA-HCV and MVA-HCV ∆C6L compared with MVA-WT include IFN-related genes (OASL, ZC3HAV1, IFN-B1, IFIT1, IFIT2, and IFIT3), genes involved in apoptosis (PMAIP1), and histones (HIST1H4D, HIST1H4F, HIST1H4K, HIST2H4B, HIST1H4H, and HIST1H4J). Additionally, in Table 1, we show the host genes that were found differentially expressed in MVA-HCV ∆C6L compared with MVA-HCV, with most of the genes downregulated by MVA-HCV ∆C6L in comparison with MVA-HCV.

To validate the microarray results, we performed real-time PCR of the mRNA samples using specific primers for three representative host genes (IFN-β, IFIT1, and IFIT 2) (Figure 3B). The results obtained were in accordance with the microarray data, showing a downregulation of these genes in MVA-HCV and MVA-HCV ΔC6L, compared with MVA-WT in the three human donor samples.

These results established that in moDCs infected with MVA-HCV and MVA-HCV ΔC6L, there is a reduced host gene expression compared with parental MVA-WT, with minor differences in extent between the two recombinant viruses.

### 3.6. MVA-HCV and MVA-HCV ΔC6L Triggered Differential Cell Recruitment than MVA-WT in the Peritoneal Cavity of Infected Mice

**Figure 4:**
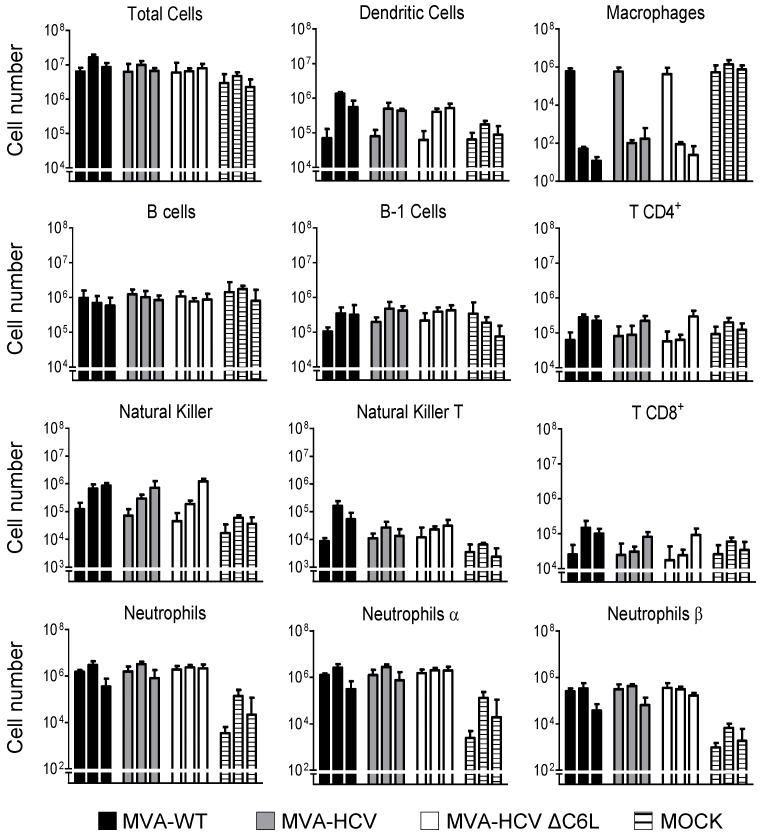
Recruitment of innate immune cells in the peritoneal cavity of infected mice. Absolute numbers of innate immune cell populations obtained from the peritoneal cavity of C57BL/6 mice infected by the i.p. route with MVA-WT, MVA-HCV, MVA-HCV ∆C6L, or PBS. Peritoneal exudate cells were collected at 6, 24, and 48 h post inoculation (first, second, and third bar in each group, respectively) from each individual mouse (*n* = 5 per group); stained for different surface markers; and absolute numbers analyzed by flow cytometry. Graphs show the logarithmic mean ± SD. Statistical significance of the comparison between MVA-HCV and MVA-HCV ΔC6L was calculated with a two-way analysis of variance (ANOVA).

As a result of the impact of MVA-HCV and MVA-HCV ΔC6L on host gene expression, we next determined the pattern of immune cells that migrate in vivo to the peritoneal cavity after the vector’s exposure. Hence, we inoculated i.p. the different viruses in five C57BL/6 mice per group (MVA-WT, MVA-HCV, MVA-HCV ΔC6L, and PBS) as described in Materials and Methods, and analyzed by flow cytometry the absolute numbers of several immune cell populations present in the peritoneal cavity at 6, 24, and 48 h post inoculation (Figure 4).

The results showed a large cell recruitment at this site for the total number of immune cells (around 10^7^ cells) following vector inoculation. At 24 h after viral infection, most of the macrophages left the peritoneal cavity, while DCs, NKs, NKT, and neutrophils (total, α and β) were highly recruited at 24 h and 48 h in MVA-WT, MVA-HCV, and MVA-HCV ΔC6L in comparison with PBS. In infections with MVA-HCV and MVA-HCV ΔC6L, there was a lower recruitment in the total number of specific immune cell types (DCs, NKs, NKT cells, and CD4^+^ and CD8^+^ T cells) than for MVA-WT at 24 h post inoculation. The comparison of MVA-HCV with MVA-HCV ΔC6L showed that both viruses behave similarly, although we observed significant differences in the cell recruitment of specific immune cells with the time (the recruitment at 6, 24, and 48 h differs from each other regardless of the virus), with MVA-HCV ΔC6L inducing a significant higher number of recruited neutrophils at 48 h (total, α and β) than MVA-HCV (* = *p* ≤ 0.05).

These results showed the impact of the MVA vectors on immune cell recruitment in the peritoneal cavity of mice during virus infection, revealing statistical differences in some innate cell recruitment between them.

### 3.7. MVA-HCV and MVA-HCV ΔC6L Induced HCV-Specific T Cell and Humoral Adaptive Immune Responses in Immunized Mice

**Figure 5:**
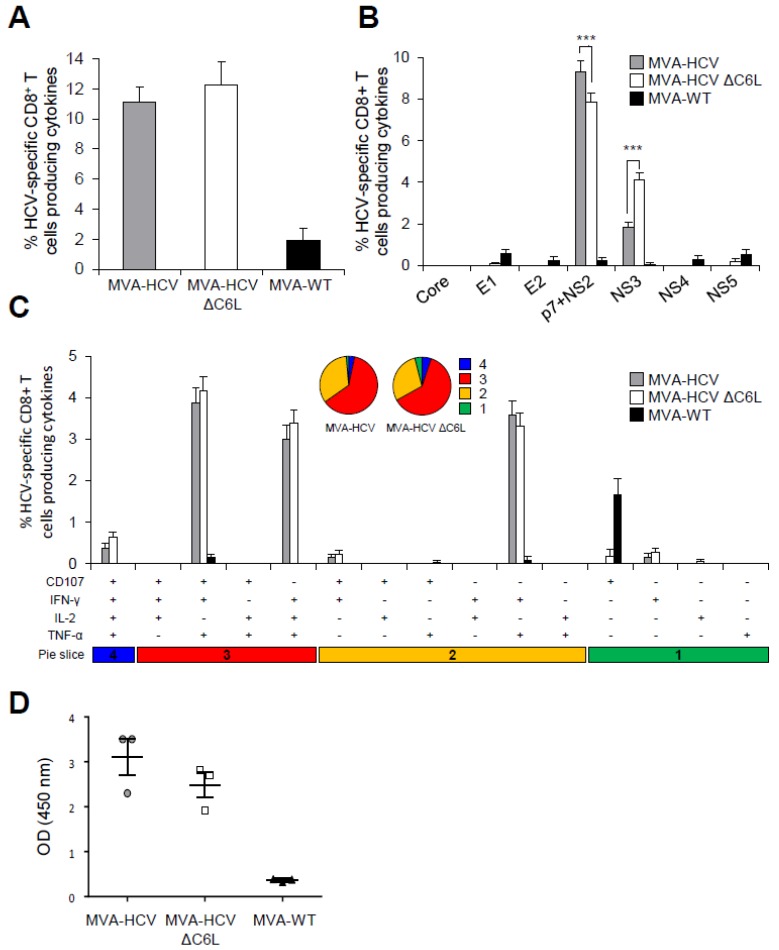
HCV-specific T cell and humoral adaptive immune responses induced in immunized mice. Splenocytes were obtained from mice (*n* = 4 per group) immunized with two doses of MVA-WT, MVA-HCV, or MVA-HCV ∆C6L 10 days after the last immunization. Then, HCV-specific CD8^+^ T cell adaptive immune responses elicited were measured by intracellular cytokine staining (ICS) after stimulation of splenocytes with different HCV peptide pools. Values from unstimulated controls were subtracted in all cases. *p* values indicate significantly response differences when comparing MVA-HCV with MVA-HCV ΔC6L (*** = *p* ≤ 0.001). Data is from one experiment representative of four independent experiments. (**A**) Magnitude of total HCV-specific CD8^+^ T cell adaptive immune responses directed against HCV antigens. The values represent the sum of the percentages of CD8^+^ T cells producing CD107a and/or IFN-γ and/or TNF-α and/or IL-2 against Core, E1, E2, p7 + NS2, NS3, NS4, and NS5 peptide pools. (**B**) Percentages of Core, E1, E2, p7 + NS2, NS3, NS4, or NS5 HCV-specific CD8^+^ T cells. Frequencies represent the sum of the percentages of CD8^+^ T cells producing CD107a and/or IFN-γ and/or TNF-α and/or IL-2 against each HCV peptide pool. (**C**) Polyfunctionality profile of total HCV-specific CD8^+^ T cell adaptive immune responses directed against HCV antigens and producing CD107a and/or IFN-γ and/or TNF-α and/or IL-2. Responses are grouped and color coded, taking into consideration the number of functions (one, two, three, or four). (**D**) Humoral immune responses induced by MVA-WT, MVA-HCV, and MVA-HCV ∆C6L against HCV E2 protein. Levels of E2-specific total IgG binding antibodies were measured by ELISA in serum from individually mice immunized with two doses of MVA-WT, MVA-HCV, or MVA-HCV ∆C6L at day 10 after the last immunization. Absorbance values (measured at 450 nm) correspond to 1/100 dilution of individual serum, and each mouse is represented by a dot. The mean ± SD are indicated.

To define whether the deletion of C6 VACV IFN-β inhibitor from MVA-HCV could have an in vivo impact on the adaptive immune response against HCV antigens, we first analyzed the HCV-specific T cell response elicited by MVA-HCV and MVA-HCV ΔC6L in mice immunized following two inoculations with each viral vector (homologous prime-boost immunization protocol) (Figure 5). Thus, eight C57BL/6 mice from each group (MVA-WT, MVA-HCV, and MVA-HCV ΔC6L) were immunized as described in Materials and Methods and half of them (*n* = 4) were sacrificed at day 10 post-boost to measure by ICS the HCV-specific adaptive T cell immune responses. Pools of splenocytes from each group were stimulated ex vivo with a panel of HCV peptide pools covering the entire sequence of HCV H77 strain (genotype 1a) and after 6 h of stimulation, cells were stained with specific antibodies to identify T cell populations (CD4 and CD8) and responding cells (CD107a on the surface of activated T cells as an indirect marker of cytotoxicity, and production of IFN-γ, TNF-α, and IL-2 cytokines).

The magnitude of the total HCV-specific CD8^+^ T cell adaptive immune responses (determined as the sum of cells producing IFN-γ, TNF-α, and/or IL-2, as well as the expression of CD107a degranulation marker) obtained for all the HCV peptide pools (Core + E1 + E2 + p7 + NS2 + NS3 + NS4 + NS5) was high and similar in mice immunized with MVA-HCV and MVA-HCV ΔC6L. The immune response was largely mediated by HCV-specific CD8^+^ T cells, while levels of HCV-specific CD4^+^ T cells were very low (Figure 5A). The profile of HCV-specific CD8^+^ T cell adaptive immune responses elicited by MVA-HCV and MVA-HCV ΔC6L immunization groups showed that the response was directed preferentially against p7 + NS2 and, to a lesser extent, toward NS3 in both immunization groups (Figure 5B). Interestingly, MVA-HCV induced significantly higher p7 + NS2-specific CD8^+^ T cell immune responses than MVA-HCV ΔC6L, while MVA-HCV ΔC6L elicited higher NS3-specific CD8^+^ T cell immune responses than MVA-HCV (Figure 5B). The quality of the HCV-specific CD8^+^ T cell immune responses was analyzed by measuring the pattern of cytokine production (IFN-γ, TNF-α, and/or IL-2) and/or its cytotoxic potential (CD107a degranulation). As shown in Figure 5C (pie charts), HCV-specific CD8^+^ T cell immune responses were similar and highly polyfunctional in animals immunized with MVA-HCV and MVA-HCV ΔC6L, with around 60% and 5% of the CD8^+^ T cells having three and four functions, respectively. HCV-specific CD8^+^ T cells producing CD107a + IFN-γ + TNF-α, IFN-γ + TNF-α + IL-2 or IFN-γ + TNF-α were the most abundant cell populations induced by MVA-HCV and MVA-HCV ΔC6L, which were of a similar magnitude between both immunization groups (Figure 5C, bars).

Next, we analyzed the humoral immune responses induced after immunization with MVA-HCV and MVA-HCV ΔC6L, quantifying, by ELISA, the total IgG levels of antibodies against HCV E2 protein (strain H77) in sera obtained from individual mice 10 days post-boost (Figure 5D). The results showed that both immunization groups were able to elicit antibodies against E2 in the adaptive phase.

These results indicate that MVA-HCV and MVA-HCV ΔC6L induced similar levels of HCV-specific CD8^+^ T cell and humoral adaptive immune responses. MVA-HCV triggered higher p7 + NS2-specific CD8^+^ T cell responses than MVA-HCV ΔC6L, while MVA-HCV ΔC6L elicited higher NS3-specific CD8^+^ T cell immune responses than MVA-HCV. Additionally, both vectors induced similar levels of antibodies against HCV E2 protein.

### 3.8. MVA-HCV and MVA-HCV ΔC6L Induced HCV-Specific T Cell Memory Immune Responses

It has been reported that memory CD8^+^ T cells are required for protection from persistent HCV [39]. Thus, to study the HCV-specific T cell memory immune responses induced by MVA-HCV and MVA-HCV ΔC6L, four mice of each immunization group were sacrificed 53 days after the second immunization and pools of splenocytes from each group were stimulated ex vivo with a panel of HCV peptide pools, similarly to the adaptive immune response protocol described above and in Materials and Methods.

**Figure 6:**
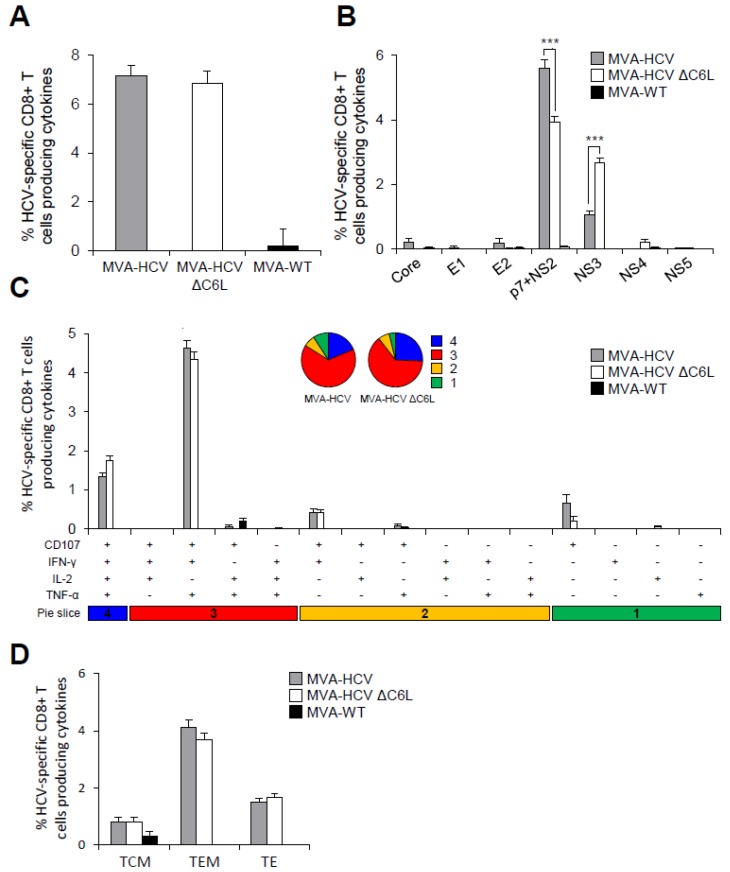
Magnitude, breath, polyfunctionality, and phenotype of HCV-specific CD8^+^ T cell memory immune responses. Splenocytes were obtained from mice (*n* = 4 per group) immunized with two doses of MVA-WT, MVA-HCV, or MVA-HCV ∆C6L 53 days after the last immunization. Then, HCV-specific CD8^+^ T cell memory immune responses elicited were measured by ICS after stimulation of splenocytes with different HCV peptide pools. Values from unstimulated controls were subtracted in all cases. *p* values indicate significantly response differences when comparing MVA-HCV with MVA-HCV ΔC6L (*** = *p* ≤ 0.001). Data is from one experiment representative of four independent experiments. (**A**) Percentage of total HCV-specific CD8^+^ T cell memory immune responses directed against HCV antigens. The values represent the sum of the percentages of CD8^+^ T cells producing CD107a and/or IFN-γ and/or TNF-α and/or IL-2 against Core, E1, E2, p7 + NS2, NS3, NS4, and NS5 peptide pools. (**B**) Percentages of Core, E1, E2, p7 + NS2, NS3, NS4, or NS5 HCV-specific CD8^+^ T cells. Frequencies represent the sum of the percentages of CD8^+^ T cells producing CD107a and/or IFN-γ and/or TNF-α and/or IL-2 against each HCV peptide pool. (**C**) Polyfunctionality profile of total HCV-specific CD8^+^ T cell memory immune responses directed against HCV antigens and producing CD107a and/or IFN-γ and/or TNF-α and/or IL-2. Responses are grouped and color coded, taking into consideration the number of functions (one, two, three, or four). Each slice in the pie charts corresponds to the proportion of the total HCV-specific CD8^+^ T cells exhibiting one, two, three, or four functions (CD107a and/or IFN-γ and/or TNF-α and/or IL-2) within the total HCV-specific CD8^+^ T cells. (**D**) Phenotypic profile of total HCV-specific CD8^+^ T cell memory immune responses directed against HCV antigens. Frequencies represent percentages of T central memory (TCM), T effector memory (TEM), and T effector (TE) HCV-specific CD8^+^ T cells producing CD107a and/or IFN-γ and/or TNF-α and/or IL-2 against HCV peptide pools. TCM, TEM, and TE phenotypes are determined on the basis of CD127 and CD62L expression: TCM (CD127^+^, CD62L^+^), TEM (CD127^+^, CD62L^−^), and TE (CD127^−^, CD62L^−^).

The magnitude of the total HCV-specific CD8^+^ T cell memory immune responses were high and similar in mice immunized with MVA-HCV and MVA-HCV ΔC6L; with the HCV-specific T cell memory immune responses largely mediated by CD8^+^ T cells, with very low HCV-specific CD4^+^ T cells (Figure 6A). Moreover, as in the adaptive phase, the pattern of HCV-specific CD8^+^ T cell memory immune responses induced by MVA-HCV and MVA-HCV ΔC6L showed that in both groups, the response was mainly directed against p7 + NS2 and then towards NS3 (Figure 6B). MVA-HCV elicited significantly higher p7 + NS2-specific CD8^+^ T cell immune responses than MVA-HCV ΔC6L, and MVA-HCV ΔC6L elicited higher NS3-specific CD8^+^ T cell immune responses than MVA-HCV (Figure 6B).

The quality of the HCV-specific CD8^+^ T cell memory immune responses showed that MVA-HCV and MVA-HCV ΔC6L triggered highly polyfunctional HCV-specific CD8^+^ T cells, with around 65% and 23% of the CD8^+^ T cells with three and four functions, respectively (Figure 6C, pie charts). HCV-specific CD8^+^ T cells producing CD107a + IFN-γ + TNF-α + IL-2 and CD107a + IFN-γ + TNF-α were the most abundant cells elicited by MVA-HCV and MVA-HCV ΔC6L (Figure 6C, bars).

Additionally, we examined the phenotype of the total HCV-specific CD8^+^ T memory cells by measuring the expression of the CD127 and CD62L surface markers, which allow the definition of the different memory subpopulations: T central memory (TCM, CD127^+^/CD62L^+^), T effector memory (TEM, CD127^+^/CD62L^−^), and T effector (TE, CD127^−^/CD62L^−^) cells (Figure 6D). The results showed that immunization with MVA-HCV and MVA-HCV ΔC6L elicited a high percentage of HCV-specific CD8^+^ T memory cells, which were mainly of the TEM phenotype, and of similar magnitude in both immunization groups.

These results indicate that there are no significant differences in the magnitude and quality of the total HCV-specific CD8^+^ T cell memory immune responses induced by MVA-HCV and MVA-HCV ΔC6L. However, the pattern of response was again different; with MVA-HCV inducing higher p7 + NS2-specific CD8^+^ T cell responses than MVA-HCV ΔC6L and MVA-HCV ΔC6L eliciting higher NS3-specific CD8^+^ T cell immune responses than MVA-HCV.

### 3.9. MVA-HCV and MVA-HCV ΔC6L Induced Similar VACV-Specific T Cell and Humoral Immune Responses

To define if the induction of specific immune responses to the HCV antigens could down-modulate the MVA vector-specific responses, we next studied the VACV-specific T cell immune responses induced by MVA-WT, MVA-HCV, and MVA-HCV ΔC6L at 10 days post-boost, following stimulation of splenocytes ex vivo with the VACV E3 peptide, similarly to the protocol described above for the analysis of the HCV-specific immune responses and in Materials and Methods.

**Figure 7:**
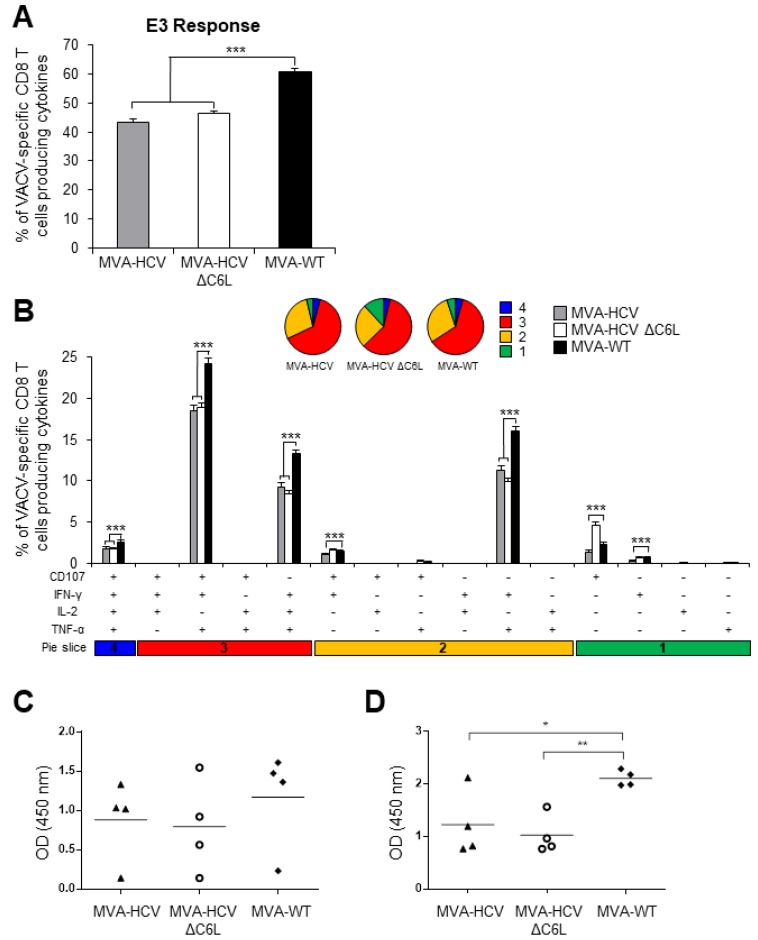
VACV-specific T cell and humoral immune responses induced in immunized mice. Splenocytes were obtained from mice (*n* = 4 per group) immunized with two doses of MVA-WT, MVA-HCV, or MVA-HCV ∆C6L at 10 days after the last immunization. Then, VACV-specific CD8^+^ T cell immune responses elicited were measured by ICS after stimulation of splenocytes with VACV E3 peptide. Values from unstimulated controls were subtracted in all cases. *p* values indicate significantly response differences (*** = *p* ≤ 0.001). Data is from one experiment representative of four independent experiments. (**A**) Magnitude of VACV-specific CD8^+^ T cell adaptive immune responses directed against VACV E3 peptide. The values represent the sum of the percentages of CD8^+^ T cells producing CD107a and/or IFN-γ and/or TNF-α and/or IL-2. (**B**) Polyfunctionality profile of VACV-specific CD8^+^ T cell adaptive immune responses directed against VACV E3 peptide and producing CD107a and/or IFN-γ and/or TNF-α and/or IL-2. Responses are grouped and color coded taking into consideration the number of functions (one, two, three, or four). (**C**,**D**) Anti-VACV humoral immune responses. Levels of anti-VACV-specific total IgG binding antibodies were measured by ELISA in serum from mice immunized with two doses of MVA-WT, MVA-HCV, or MVA-HCV ∆C6L at day 10 (**C**) and at day 53 (**D**) after the last immunization. Absorbance values (measured at 450 nm) correspond to 1/100 dilution of individual serum, and each mouse is represented by a dot. The mean ± SD are indicated. *p* values indicate significantly response differences (* = *p* ≤ 0.05; ** = *p* ≤ 0.01).

The magnitude of the VACV-specific CD8^+^ T cell immune responses induced by MVA-HCV and MVA-HCV ΔC6L were similar, but significantly lower than the response induced by MVA-WT (Figure 7A). Analysis of the quality of the VACV-specific CD8^+^ T cell immune responses showed that although MVA-HCV and MVA-HCV ΔC6L triggered lower magnitude than MVA-WT, in the three immunized groups, VACV-specific CD8^+^ T cells were highly polyfunctional, with around 70% of the CD8^+^ T cells with three and four functions (Figure 7B).

The humoral immune responses induced after immunization with MVA-WT, MVA-HCV, and MVA-HCV ΔC6L was quantified by ELISA, measuring the total IgG levels against VACV observed in serum obtained from individual mice at 10 and 53 days post-boost (Figure 7C,D, respectively). The results showed that MVA-HCV and MVA-HCV ΔC6L elicited similar levels of anti-VACV antibodies, but at lower levels than those induced by MVA-WT.

These results showed that the CD8^+^ T cell and humoral immune responses to the virus vector are similarly induced between MVA-HCV and MVA-HCV ΔC6L, but the levels are lower than those induced by the parental vector MVA-WT, suggesting some immune down-regulatory effects of HCV antigens over the poxvirus vector.

## 4. Discussion

In spite of the great advances in the development of effective anti-HCV drugs, and because of a worldwide large population of infected persons, undoubtedly there is the need to develop an HCV vaccine. An ideal vaccine against HCV should be able to cure the disease during primary infection and prevent chronicity, because HCV-associate diseases are mostly manifested during chronic infection where treatment is less successful and liver is being severely damaged [40]. Spontaneous cure of infection happens in 30% of infected people, resulting in a complete viral clearance and higher chances of resolving a reinfection, staying away from chronicity. Therefore, it is clear that some components of the immune system are able to skew the outcome of HCV infection towards resolution after the acute phase, making this the main goal of a successful HCV vaccine [11].

Over the last few years, several HCV vaccine candidates aimed to induce strong humoral responses and/or T cell responses have been tested in preclinical studies, but only a few of them reached clinical trials [40]. Several viral vector-based vaccines have been tested in humans, including adenovirus vectors expressing the nonstructural HCV proteins NS3, NS4, and NS5mut (genetically inactivated polymerase gene) (Ad6NSmut and AdCh3NSmut) in phase I clinical trials (study HCV001 NCT01070407 in healthy volunteers and study HCV002 NCT01094873 in chronically infected patients), showing that both vectors are safe and immunogenic [41]. Furthermore, an MVA expressing the same antigens (MVA-NSmut) was also assessed alone or in combination with Ad6NSmut (NCT01701336 in chronically infected patients) or AdCh3NSmut (NCT01296451 in healthy volunteers and HCV infected patients) in phase I clinical trials [42]. Currently, AdCh3NSmut and MVA-NSmut are being tested in a phase I/II clinical trial (NCT01436357 in uninfected injection drug users, final data collection in July, 2018). Lastly, an MVA vector encoding NS3, NS4, and NS5B (TG4040) was studied in a dose-escalating phase I clinical trial (NCT00529321 in untreated chronic patients), showing that this vector is also safe and immunogenic. TG4040 was further tested in a phase II clinical trial as a therapeutic vaccine combined with standard of care treatment (PEG-IFN-α with ribavirin) (NCT01055821 in untreated chronic patients) [43]. In clinical and preclinical trials, both adenoviruses and MVA vectors elicited HCV-specific CD8^+^ T cell responses; however, because of the anti-adeno sero-prevalence in human population and the cross-reactive immunity between both vectors (AdCh3NSmut and Ad6NSmut), the immunogenicity was improved when MVA was administered as a boost [42]. Moreover, a recent study showed that in immunized mice, an MVA vector expressing NS3/4A is superior to adenovirus-5 vector in the induction of CD8^+^ T cell memory responses against HCV [44].

We have previously described an MVA-based vaccine candidate for HCV (termed MVA-HCV) that expresses most of the HCV genome (Core, E1, E2, p7, NS2, NS3, NS4A, NS4B, NS5A, and part of NS5B protein) [26]. MVA-HCV elicited polyfunctional T cell responses with memory phenotype in wild-type and humanized mice, being the first vaccine candidate encoding all HCV proteins (structural and nonstructural), and thus covering most of the T and B cell determinants described for HCV. However, efforts to understand the HCV-specific immune responses elicited by MVA-HCV are needed in order to improve this vaccine candidate. Firstly, despite its attenuated phenotype, MVA still encodes for proteins that can interfere with host immune responses to viral infection [45,46,47], and it is described that deletion of VACV immunomodulatory genes can enhance its immunogenicity [20]. Secondly, IFN-based therapy in combination with ribavirin has been the first treatment of choice, despite its well-known side effects, and even in the era of new DAA therapy, treatment with IFN results in improvement of response rates and higher sustained virological response in genotypes 1 [48,49], 2, and 3 [50,51]. Therefore, IFN release and a strong immune response are critical in the control and resolution of HCV infection, providing signals for the efficient priming of the adaptive branch of immune response [19].

Hence, in an effort to define the immunomodulatory role of an IFN response over the MVA-HCV vaccine candidate, we used here the same strategy previously shown to enhance HIV-1-specific cellular and humoral responses in the MVA-B vaccine [22,24]. Thus, we deleted the VACV *C6L* gene (encoding a type I IFN inhibitor protein that targets the TBK1-IKKe complex to inhibit IRF3 and IRF7 activation) from MVA-HCV to generate a novel HCV vaccine candidate termed MVA-HCV ΔC6L. The vector MVA-HCV ΔC6L efficiently produces all HCV antigens (Core, E1, E2, p7, NS2, NS3, NS4A, NS4B, NS5A, and a part of NS5B) at the same level as MVA-HCV during the course of virus infection. MVA-HCV ΔC6L replicates similarly to MVA-HCV in permissive culture cells, indicating that deletion of *C6L* had no effect in viral kinetics. Unexpectedly, in infected human macrophages or moDCs, MVA-HCV ΔC6L did not increase the expression of type I IFN or IFN-related genes compared with parental MVA-HCV. Although both vectors downregulated host gene expression in comparison with MVA-WT, they still induced immune activation despite the presence of HCV proteins, which are potent innate response inhibitors, especially the NS3 HCV protein [52,53].

Microarray analyses in infected human moDCs provided an identification of the host genes triggered in response to the HCV proteins expressed from MVA-HCV and MVA-HCV ∆C6L. Under stringent conditions of microarray analysis, the results showed that MVA-HCV and MVA-HCV ∆C6L differentially downregulate IFN-related genes (OASL, ZC3HAV1, IFN-B1, IFIT1, IFIT2, and IFIT3), genes involved in apoptosis (PMAIP1), and histones (HIST1H4D, HIST1H4F, HIST1H4K, HIST2H4B, HIST1H4H, and HIST1H4J) in comparison with MVA-WT. This downregulation of type I IFN and IFN-related genes confirmed the previous RT-PCR results in infected moDCs, and is in accordance with the role exerted by the HCV proteins, especially NS3, inhibiting type I IFN signaling [52,53]. In a different virus-cell system, in which the same HCV cassette was expressed from a transcriptional regulated replication competent VACV-based delivery system (VT7-HCV7.9) in HeLa cells, we previously reported the induction of cell apoptosis by HCV proteins mediated by IFN-induced enzymes PKR and RNase-L [54], and that HCV protein expression modulated transcription of genes associated with lipid metabolism, oxidative stress, apoptosis, and cellular proliferation [55]. Down-regulation of histone mRNA levels, which are tightly regulated during DNA replication, indicates that cell proliferation and cell cycle progression are likely impaired in cells infected with MVA-HCV and MVA-HCV ΔC6L. In this regard, it has been reported that infection of Huh7.5 cells with HCV resulted in inhibition of histone H4 methylation/acetylation and histone H2AX phosphorylation, with a significant change in expression of genes important for hepatocarcinogenesis, inhibiting DNA damage repair [56].

In cells recruited in the peritoneal cavity of mice inoculated with MVA-HCV, MVA-HCV ∆C6L, MVA-WT, or PBS, we also observed an inhibitory effect in the innate immune responses induced by the MVA vectors expressing HCV proteins, which resulted in less recruitment of specific innate immune cell types, such as DCs, NK, NKT, CD4^+^, and CD8^+^ T cells at 24 h post-inoculation in comparison with MVA-WT. This lower recruitment of innate immune cells is in accordance with the downregulation of type I IFN and IFN-related genes observed in human moDCs infected with MVA-HCV and MVA-HCV ∆C6L.

We have previously shown that deletion of VACV *C6L* gene in the vector backbone of the MVA-B vaccine candidate against HIV/AIDS significantly upregulates IFN-β and IFN-α/β-inducible genes [22,24]. Here, the same MVA vector, except that it expressed HCV instead of HIV-1 antigens, shows opposite results, likely because of the nature of HCV proteins in inhibiting type I IFN responses. This result highlights the influence of expressing different heterologous antigens in the biology of the MVA vector. It has been described that nonstructural proteins from the *Flaviviridae* family interfere with the host immune responses to facilitate viral propagation [57]: HCV NS2 inhibits IRF3 phosphorylation, and thus the production of IFN-β, IFNα1, IFNλ1, IFNλ3, and chemokines CCL5 and CXCL19 [58]; HCV NS3/4A blocks TLR3 signaling pathway [53], IRF3 phosphorylation [59], and inhibits RLR signaling [52,53]; HCV NS4B avoids STING accumulation, suppressing RLR signaling [60]; and HCV NS5A impairs TLR-MyD88 signaling [61], blocks IRF7 nuclear translocation [62], and binds to JAK/STAT [63] and PKR [64] inhibiting their signaling pathway. Thus, in the context of the MVA-HCV vaccine candidate, production of all HCV proteins negatively impacts host gene expression, and deletion of the VACV C6 type I IFN inhibitor is not enough to counteract and reverse the strong IFN inhibition exerted by the HCV proteins; therefore, an increase in IFN-β and IFN-α/β-inducible genes is not obtained.

Despite the inhibition of type I IFN exerted by infection with MVA-HCV and MVA-HCV ∆C6L, these vectors are still able to induce HCV-specific T and B cell responses in immunized mice. Several studies have proven that a potent T cell response is important for virus clearance [65], with monospecific HCV-specific CD8^+^ T cell responses being associated with viral persistence [66]. Hence, a vaccine against HCV should ideally contain most of the HCV antigens, including structural and nonstructural proteins, in order to elicit T cell responses that can target most of the HCV epitopes. As shown here, MVA-HCV ∆C6L elicited high and polyfunctional HCV-specific CD8^+^ T cells at a similar level to MVA-HCV, but the specificity of the response against the HCV antigens was different, with MVA-HCV ∆C6L inducing a significantly higher NS3-specific response, while MVA-HCV elicited higher p7 + NS2-specific response. The impact of these differences needs to be further studied, but it has been proposed that a response against HCV NS3 protein could be important in protection because of the role of this protein in HCV infection, with several viral epitopes triggering CD8^+^ T cell responses [67,68]. Moreover, DCs pulsed with HCV NS3 protein induced immune responses and protection from infection in mice inoculated with recombinant VACV expressing NS3 [69], and NS3-specific CD8^+^ T cell responses, were observed in a chimpanzee clearing HCV infection [70], reinforcing the important role of NS3-specific responses in HCV. Additionally, both MVA-HCV and MVA-HCV ∆C6L induced similar proportions of HCV-specific CD8^+^ T memory cells, with a predominance TEM phenotype, which has been described to be required to eliminate the virus through the production of cytolytic molecules and cytokines [71]. Furthermore, MVA-HCV and MVA-HCV ∆C6L induced similar levels of VACV vector-specific CD8^+^ T cell and humoral immune responses, but these responses were significantly lower than those induced by the parental MVA-WT. These findings further support the immune suppressive effects mediated by the HCV antigens. Moreover, the reduced levels of anti-vector antibodies have the added advantage of the potential delivering of multiple boosting doses to the animals with the MVA-HCV vectors.

To boost the efficacy of an HCV vaccine candidate, T and B cell responses should be combined in a synergistic effect [40], because there is rising evidence that antibodies can be necessary to control HCV replication, especially in early stages of infection [1,8,12], and it has been described that passive transfer of anti-E2 antibodies protects against infection in chimpanzees and chimeric mice [72,73,74]. Here, it is shown for the first time that MVA-HCV and MVA-HCV ∆C6L are able to elicit in immunized mice antibodies against E2 protein, the major target for neutralizing antibodies [75]. Thus, because any effective vaccine should induce both T and B cell immunity in an optimized regimen, our HCV vaccine candidates fulfill these requirements and are able to induce high levels of HCV-specific T cell and humoral immune responses.

Overall, the results show that production of HCV proteins in moDCs infected with MVA-HCV and MVA-HCV ΔC6L modulates expression of host genes involved in innate immunity, decreasing the levels of type I IFN. In infected mice, both MVA-HCV and MVA-HCV ∆C6L induced high and polyfunctional HCV-specific CD8^+^ T cell responses and humoral responses against HCV antigens, making it a good vaccine candidate against HCV. Our results yield valuable cellular and immunological information about the use of MVA as a vaccine candidate against HCV that could be tested, alone or combined with other immunogens, in future prophylactic and/or therapeutic clinical trials against HCV.

## Figures and Tables

**Table 1:** List of genes differentially regulated in human monocyte-derived dendritic cells (moDCs) infected with modified vaccinia virus Ankara (MVA)-hepatitis C virus (HCV) ∆C6L in comparison with MVA-HCV. ^a^ The gene name and the probe ID are indicated. ^b^ Differentially expressed genes were evaluated by the non-parametric algorithm “Rank Products” available as “RankProd” package at Bioconductor, and the genes were filtered using a *p* value Limma < 0.05 and a fold change of ≥2 or ≤−2.

Gene Name (Probe identity (ID)) ^a^	Accession Number	Fold Change ^b^
Transmembrane protein 11 (TMEM11)	NM_003876	+2.05
KIAA0586 (KIAA0586)	NM_014749	+2.00
CD28 molecule (CD28)	NM_006139	−2.00
FLJ30313 (lnc-GATA5-2)	lnc-GATA5-2:2	−2.01
Aquaporin 1 (AQP1)	NM_198098	−2.03
Gessler Wilms tumor Homo sapiens (SMCR2)	AI821758	−2.05
Oogenesis homeobox (NOBOX)	NM_001080413	−2.05
RAS oncogene family (RAB6A)	NM_002869	−2.08
Ribosomal protein S28 (RPS28)	NM_001031	−2.08
Major histocompatibility complex, class II, DR beta 5 (HLA-DRB5)	NM_002125	−2.09
Chimerin 2 (ENST00000409922)	ENST00000409922	−2.10
Nuclear factor of activated T-cells, cytoplasmic, calcineurin-dependent 1 (NFATC1)	NM_172390	−2.13
RAS guanyl releasing protein 2 (RASGRP2)	ENST00000377494	−2.14
Glycosyltransferase 3 family (UGT3A1)	NM_152404	−2.16
Myotubularin related protein 3 (MTMR3)	NM_021090	−2.17
Keratin associated protein 2-1 (KRTAP2-1)	NM_001123387	−2.18
keratin associated protein 5-4 (KRTAP5-4)	NM_001012709	−2.19
G protein-coupled receptor 88 (GPR88)	NM_022049	−2.19
G protein pathway suppressor 2 pseudogene (ENST00000430745)	ENST00000430745	−2.19
Secretory trafficking family member B (MON1B)	NM_014940	−2.21
Solute carrier family 16, member 11 (SLC16A11)	NM_153357	−2.24
Vacuolar protein sorting 18 homolog (VPS18)	NM_020857	−2.26
Synapse defective 1, Rho GTPase, homolog 2 (SYDE2)	NM_032184	−2.27
Neuron-derived neurotrophic factor (NDNF)	NM_024574	−2.30
Homeobox 1 (EMX1)	BC037242	−2.32
Actin-like protein 3 (THC2499666)	THC2499666	−2.32
Interleukin 15 receptor (IL15RA)	ENST00000379971	−2.39
Chemokine (C-C motif) ligand 18 (CCL18)	NM_002988	−2.40
cDNA clone NT2RI2025693 (ENST00000587777)	ENST00000587777	−2.42
Cellular repressor of E1A-stimulated genes 1 (CREG1)	NM_003851	−2.46
NYN domain and retroviral integrase containing (NYNRIN)	NM_025081	−2.49
Uncoupling protein 3 (UCP3)	NM_022803	−2.53
Phospholipase C (PLCH2)	NM_014638	−2.57
Long intergenic non-protein coding RNA 687 (LINC00687)	NR_110635	−3.05
Synuclein, beta (SNCB)	NM_001001502	−3.05
Arylformamidase (AFMID)	NM_001145526	−3.20
